# Synthesis and Plasmonic Chiroptical Studies of Sodium Deoxycholate Modified Silver Nanoparticles

**DOI:** 10.3390/ma11081291

**Published:** 2018-07-26

**Authors:** Jing Wang, Kai-Xuan Fei, Xin Yang, Shuai-Shuai Zhang, Yin-Xian Peng

**Affiliations:** School of Environmental and Chemical Engineering, Jiangsu University of Science and Technology, Zhenjiang 212003, China; fei1567552@gmail.com (K.-X.F.); y122821@gmail.com (X.Y.); zhang7793622@gmail.com (S.-S.Z.)

**Keywords:** plasmonic chirality, Ag nanoparticles, sodium deoxycholate

## Abstract

Sodium deoxycholate modified silver nanoparticles prepared in the presence of sodium deoxycholate as a chiral inducer exhibit plasmonic circular dichroism (CD) signals. The plasmon-induced chirality arises from the presence of chiral molecules (sodium deoxycholate) on the surface of Ag nanoparticles, which transfer their chiral properties to the visible wavelength range due to the Coulomb interactions between the chiral molecules and plasmonic nanoparticles. The prepared Ag nanoparticles (NPs) exhibit distinct line shapes of plasmonic CD, which can be tailored by varying the pH values of the solutions. A mechanism was proposed to explain the generation of the distinct plasmonic CD shapes, which indicated that the arrangements of chiral molecules in the plasmonic hot spots between Ag NPs are crucial for the induced plasmonic CD.

## 1. Introduction

Noble metal nanoparticles (NPs) display unique optical properties due to their unusual surface plasmon resonances (SPR) in the visible region, which makes them particularly promising for applications in optical devices [1,2] and the biomedical field [3,4]. Surface modification of plasmonic NPs with chiral organic molecules could create optical responses in the visible spectral range. Chiral noble metal nanomaterials have attracted great interest in recent years because of their potential applications in chiral catalysis [5,6], chiral discrimination [7,8], and high-sensitive detection [9,10,11].

Chiral templates such as DNA helical nanostructures [12,13,14,15] and chiral supramolecular assemblies [16,17,18] are widely utilized to prepare plasmonic noble metal NPs with chiroptical response in the visible region. Another choice for the fabrication of chiral noble metal NPs is to use low-molecular-mass chiral thiols, including cysteine, glutathione and penicillamine as chiral inducers. It has been well demonstrated that chiral thiol protected noble metal nanoclusters have a chiral perturbation placed upon their metal-based electronic transitions and consequently display chiroptical activity. Recently, cysteine was utilized to synthesize chiral plasmonic silver nanoparticles that exhibit optical activities at the wavelengths of SPR of Ag NPs [19]. Optical activities were observed after modification of Ag colloids with cysteine, a thiol-containing amino acid. Wang and co-workers reported that plasmon-induced circular dichroism (CD) signals in the visible region are observed for gold nanosphere clusters with cysteine molecules locating at the hot spots of the linear clusters [20]. The observed plasmon-induced CD responses show a significant correlation with the chiral nature of molecules at the hot spots (the enhancement of the electromagnetic field occurring at the nanogaps between the nanoparticles). It still remains a great challenge to use chiral small molecules without thiol group as capping agents to prepare noble metal NPs with tunable plasmonic chiroptical response.

Herein, we report on the plasmonic chiroptical responses of silver NPs prepared with a chiral small molecule without thiol group, sodium deoxycholate (NaDC), as the inducing agent. The as-synthesized NaDC-modified Ag NPs display optically tunable plasmonic CD features by varying the pH values of the reaction mixtures. The Ag NPs prepared at high pH are arranged into short linear chains, exhibiting a strong positive Cotton effect at the SPR region. For Ag NPs prepared at low pH values, split plasmonic CD lines are achieved accompanied by the apparent changes in the absorption spectra. It is suggested that the arrangements of chiral capping agents in the plasmonic hot spots between the adjacent Ag NPs is responsible for the variations of the CD signals.

## 2. Materials and Methods

### 2.1. Materials

Silver nitrate (AgNO_3_), sodium deoxycholate (NaDC), deoxycholic acid (HDC), sodium citrate, and sodium borohydride (NaBH_4_) were obtained from Sigma-Aldrich (Shanghai, China) and used without further purification. Deionized water was used for all the experiments.

### 2.2. In Situ Preparation of NaDC-Capped Ag NPs

The synthesis of NaDC-modified Ag NPs was achieved by the reduction of the mixed solution of AgNO_3_ and NaDC. In a typical synthesis, 100 μL of sodium deoxycholate solution (20 mM) and 100 μL of AgNO_3_ solution (10 mM) were added to 4.8 mL of water and reacted for 30 min under magnetic stirring (IKA-Werke GmbH & Co.KG, Guangzhou, China). Then 100 μL of NaBH_4_ solution (10 mM) was added into the mixed solution of AgNO_3_ and NaDC under vigorous stirring. The samples with other molar ratios of AgNO_3_ to NaDC were prepared in the same way by varying the volume of silver nitrate or NaDC solution added. The pH values of the reaction mixtures were adjusted by using solutions of sodium hydroxide and hydrochloric acid.

### 2.3. Preparation of NaDC-Capped Ag NPs by Ligand Exchange Reaction

Sodium citrate-stabilized Ag nanoparticles were synthesized by the reduction of the mixed solution of AgNO_3_ and sodium citrate. 400 μL of AgNO_3_ solution (50 mM) and 200 μL of sodium citrate solution (300 mM) were added to 18.4 mL of water. Then 500 μL of NaBH_4_ solution (50 mM) was added into the reaction mixture under vigorous stirring. The prepared sodium citrate-modified Ag nanoparticles were subsequently modified with NaDC molecules by adding NaDC solution to the citrate-stabilized Ag NPs. A solution of 120 μL of NaDC (2 mM) was added to 0.75 mL of sodium citrate-stabilized Ag NPs and the obtained mixture was aged for half an hour. Then NaDC-modified Ag NPs prepared by ligand exchange reaction were collected by centrifugation (Changcheng instruments Co., Ltd, Zhengzhou, China) at 12,000 rpm for 20 min and the precipitate was redispersed in 1.5 mL of water.

### 2.4. Preparation of Ag NPs in NaDC and HDC Mixed Solutions

In a typical synthesis, 50 μL of sodium deoxycholate solution (0.2 mM), 30 μL of deoxycholic acid in ethanol solution (5 mM), and 100 μL of AgNO_3_ solution (3 mM) were added to 3 mL of water and reacted for 1 h under magnetic stirring. Then 100 μL of NaBH_4_ solution (3 mM) was added into the reaction mixture under vigorous stirring. The samples with other molar ratios of NaDC to HDC were prepared in the same way by varying the volume of NaDC or HDC solution added.

### 2.5. Ex Situ Preparation of NaDC-Capped Ag NPs under the Acidic Condition

0.25 mL of H_2_O was added to 0.75 mL of sodium citrate-stabilized Ag nanoparticles. This was followed by addition of 75 μL of NaDC (10 mM) and the obtained mixture was aged for half an hour. Then 200 μL of HCl solution (1.2 mM) was added to the above mixture.

### 2.6. Characterization

UV-vis absorption spectra of the silver NPs prepared were recorded using a U-3010 spectrophotometer (Hitachi Ltd., Tokyo, Japan) at room temperature. CD spectra were measured with a JASCO J-1500 CD (Japan Spectroscopic Co., Ltd., Tokyo, Japan) spectropolarimeter using a quartz cell of 1 cm optical path length. Transmission electron microscopy (TEM) images were taken on a JEM-2010 (Japan Electron Optics Laboratory Co., Ltd., Tokyo, Japan) electron microscope operated at an acceleration voltage of 120 kV. The sample was dropped onto a carbon-coated copper grid and allowed to dry at room temperature before being analyzed.

## 3. Results and Discussions

### 3.1. Chiroptical Property of In Situ Prepared NaDC-Capped Ag NPs at Neutral pH

**Figure 1:**
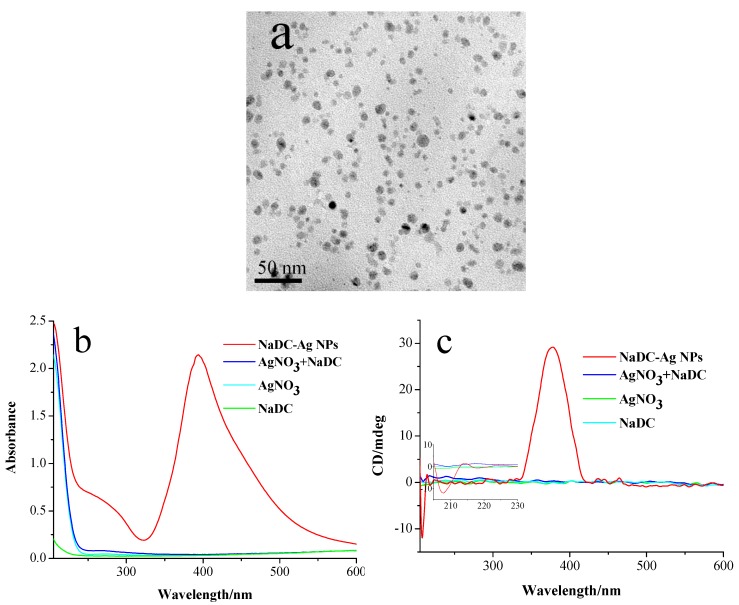
(**a**) TEM image of NaDC modified Ag NPs prepared at pH 7.0 by reducing AgNO_3_ with NaBH_4_ in the presence of NaDC; (**b**) UV-vis absorption and (**c**) CD spectra of NaDC modified Ag NPs prepared at pH 7.0, AgNO_3_, NaDC, the mixture of AgNO_3,_ and NaDC. The inset of (**c**) shows the magnified spectra in the wavelength region of 200–230 nm. The concentration of NaDC was 0.4 mM, and its molar ratio to AgNO_3_ was 2.

Sodium borohydride (NaBH_4_) was added to the mixed solution of sodium deoxycholate (NaDC) and AgNO_3_ which leads to the formation of Ag NPs modified by chiral NaDC molecules as seen in the TEM image (Figure 1a). The prepared silver NPs are essentially spherical in shape with average particle sizes of 7.5 ± 2.5 nm (Figure S1 in Supplementary Materials). They are arranged to form short linear chains which are caused by the self-assembling of Ag NPs mediated by NaDC molecules. The inter-particle distance, d, (the distance between the neighboring particles) in the assemblies is approximately 2.5 ± 1.1 nm (Figure S2 in Supplementary Materials). Figure 1b,c display the absorbance and CD spectra of NaDC, AgNO_3_, their mixture and the formed Ag NPs. The as-synthesized Ag NPs in the UV-vis spectrum displays an intense peak centered at 390 nm (Figure 1b) in the surface plasmon resonance (SPR) region. Figure 1c shows the CD spectrum of formed NaDC-modified silver NPs after NaBH_4_ was added to the mixture solution of NaDC and AgNO_3_, exhibiting a strong positive Cotton effect at 380 nm, which is associated with the SPR absorption of the silver NPs [21,22]. Before NaBH_4_ was added to the mixture solution, the solution did not show any CD signal in the UV region. Due to the sensitivity limitation of the instrument, the CD signature of NaDC at the concentration of 0.4 mM is not observable in the CD spectrum (Figure 1c). Observable CD signals from chiral NaDC require a significantly higher concentration (Figure S3 in Supplementary Materials). It should be noted that the CD signal of NaDC molecules that is absent in the mixture of NaDC and AgNO_3_ solution before reduction appears in the prepared NaDC modified silver NPs (see the inset of Figure 1c). Theoretical calculations demonstrate that the chiral property of a molecule can be both strongly enhanced and transferred to the visible wavelength by using a plasmonic hot spot in a nanoparticle dimer [23]. The mechanism of plasmonic CD comes from the Coulomb interaction, which is greatly amplified in a plasmonic hot spot. Metal NPs’ assemblies are expected to have hot plasmonic spots at gaps between nanoparticles. In our case, NaDC-modified silver NPs are arranged into short assemblies where hot spots appear between the adjacent Ag NPs, which greatly enhances the chiral properties of NaDC molecules located at the gaps and facilitates the transferring of the CD signal from the UV to the SPR region of Ag NPs.

### 3.2. Chiroptical Property of Ex Situ Prepared NaDC-Capped Ag NPs

**Figure 2:**
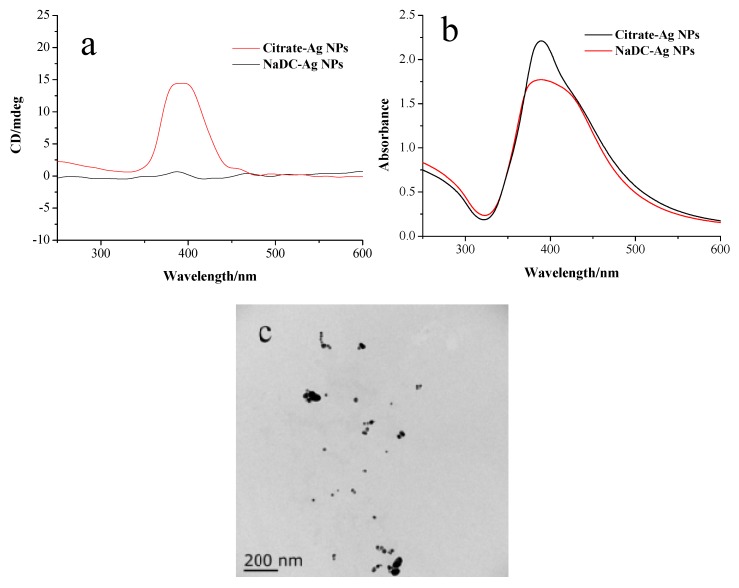
(**a**) CD and (**b**) UV-vis absorption spectra of sodium citrate-modified Ag NPs and NaDC-modified Ag NPs. (**c**) TEM image of NaDC-modified Ag NPs prepared by ligand exchange reaction after the centrifugation.

In order to clarify the origin of the observed optical activity, whether the metal core is intrinsically chiral or the optical activity is induced by the chiral environment, silver NPs were prepared in an achiral environment in the presence of sodium citrate as the stabilizer. The sodium citrate-modified silver nanoparticles were then incubated with NaDC solutions. During the process of incubation, the NaDC molecules can be chemisorbed through carboxylate groups onto the surface of the silver particles by the ligand exchange reactions. After the incubation, NaDC-modified Ag NPs were collected by centrifugation and redispersed in water. Figure 2a exhibits the CD signal of NaDC-modified Ag NPs obtained by ligand exchange reactions, showing a strong positive Cotton effect at 390 nm. In contrast to ex situ prepared NaDC-modified Ag NPs, sodium citrate-modified silver particles did not exhibit any CD signal in the SPR region. Figure 2b indicates that the absorption peaks of Ag NPs become broader after NaDC modification, suggesting that NaDC modified Ag NPs slightly aggregate. This is confirmed by the TEM images of NaDC modified Ag NPs containing dimers and linear chain assemblies (Figure 2c). It has been reported that the plasmonic CD signals can be enhanced in aggregates composed of chiral molecule-metal nanoparticle conjugates, which creates hot spots between Ag NPs and remarkably amplifies the electromagnetic fields [24]. The mechanism of plasmonic CD signals is based on the Coulomb interaction between chiral molecules and plasmonic modes in the hot spot.

Note that the CD spectrum of NaDC-modified Ag NPs prepared ex situ (by the ligand exchange reactions) is very similar to that of in situ prepared NaDC-modified Ag NPs. If the chiral core is the main origin, core rearrangements will yield the possibility of changes in the CD signatures. Similar spectra seem to contradict a chiral core model, indicating that the metal core of silver NPs prepared in situ is not intrinsically chiral. The origins of optical activity of chiral molecule-capped metal nanoparticles have been discussed experimentally and theoretically. Three major mechanisms have been proposed to explain the observed optical activity: (a) The optical activity arises from an intrinsically chiral inorganic core. The presence of chiral ligands can favor the growth of one of the two possible enantiomers of the core [25]; (b) for the achiral inorganic core, optical activity is induced by a chiral organic shell through vicinal effects or through a chiral electrostatic field [26,27]; and, (c) for an achiral core, the relaxation of the surface atoms involved in the adsorption of the chiral ligand creates a chiral footprint (i.e., a chiral surface-atom distortion caused by at least a double interaction) [28,29]. Based on our experimental results, scheme (a) is unlikely for NaDC-modified silver NPs. Gautier and Bürgi proposed a chiral footprint model to explain the observed optical activity for gold NPs covered with N-isobutyryl-l/d-cysteine [28]. It was suggested that the surface chiral distortion caused by a double interaction through the adsorption of both carboxylate groups and thiolates was the origin. The NaDC molecule contains one carboxyl and two hydroxyl groups, which can be chemisorbed onto silver’s surface. Unlike the sulfhydryl group, the interactions between carboxylate group (or hydroxyl groups) and Ag NPs are not very strong. It seems unlikely that NaDC molecules can distort the Ag atoms on the surface of Ag NPs.

A theory presented by Govorov et al. demonstrated that a chiral molecule coupled with a non-chiral metal NP can transfer its chiral property to the visible wavelength range [30]. The optical responses at the plasmon frequency are associated with Coulomb interactions between the chiral molecule and non-chiral plasmonic nanoparticles. Another theoretical study showed that a chiral molecule placed in a plasmonic hot spot can induce the enhanced plasmonic CD signal [31]. The enhancement originates from the giant enhancement of the electric field at the chiral molecule. These theories can be applied to our system. After the ligand exchange reactions, chiral molecules are chemisorbed on the surfaces of Ag NPs via carboxylate groups. NaDC is an amphiphilic molecule, which consists of a convex hydrophobic surface (the steroid ring) and a concave hydrophilic surface (hydroxyl groups and carboxylate ion). NaDC molecules on the surfaces of Ag NPs have the tendency to self-assemble via hydrophobic interactions, which promotes the aggregation of Ag NPs. The hot spots appear in the small aggregates of Ag NPs, which greatly amplifies the plasmonic CD signal.

### 3.3. Chiroptical Properties of Prepared NaDC-Capped Ag NPs under the Acidic Conditions

**Figure 3:**
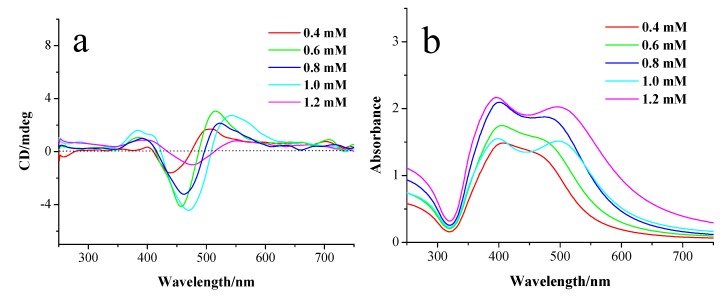

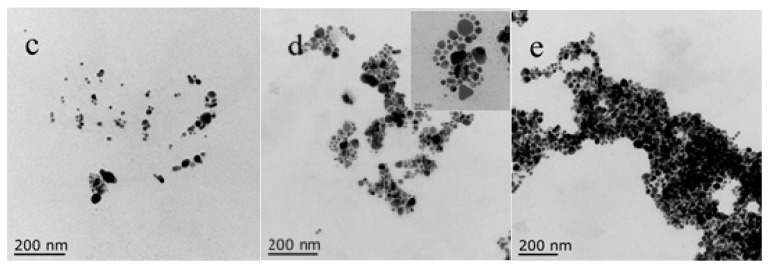
(**a**) CD and (**b**) UV-vis absorption spectra of Ag NPs at pH 5.2 with AgNO_3_ concentrations of 0.4, 0.6, 0.8, 1.0, and 1.2 mM, respectively. (**c**–**e**) TEM images of Ag NPs prepared at pH 5.2 with AgNO_3_ concentrations of 0.4, 0.6 and 1.0 mM, respectively. The inset of (**d**) shows the amplified TEM image of Ag NPs prepared at pH 5.2 with AgNO_3_ concentrations of 0.6 mM.

The shapes of the CD spectra can be altered by reducing the pH values of the reaction solutions. When the pH value of the solution is reduced to below the pK_a_ of NaDC (approx. 6.29) [32], distinct CD signals are observed at the SPR region for the prepared Ag NPs, as shown in Figure 3a. The CD spectrum of the silver NPs prepared at pH 5.2 exhibits a splitting CD signal with two positive Cotton effects at 390 nm and 543 nm, and a negative Cotton effect at 469 nm (Figure 3a), which is distinct from those of Ag NPs prepared at a pH value higher than the pK_a_ of NaDC. The characteristic splitting of the CD signature of silver NPs prepared at a pH value lower than the pK_a_ of NaDC strongly suggests the existence of plasmonic dipole-dipole interactions between adjacent Ag NPs [33,34]. The absorption of silver NPs prepared at pH 5.2 shows two peaks at 398 and 500 nm (Figure 3b). The appearance of the new peak at the longer wavelength indicates that the aggregates of Ag NPs form in the solution at the low pH value. The lowering of the pH values of the solution causes HDC to be generated by the neutralization of NaDC. The obtained HDC molecules interact with NaDC molecules to form associations through hydrophobic interactions and hydrogen bonds, which facilitate the aggregation of Ag NPs.

The influence of the concentrations of AgNO_3_ on the chiroptical activities of the prepared Ag NPs was investigated. For the Ag NPs prepared at pH 5.2, their absorption spectra exhibit two split absorption peaks that are caused by the coupling between Ag NPs in the aggregates (Figure 3b), which can be confirmed by TEM images (Figure 3c–e). The peak appearing at the longer wavelength experiences a redshift accompanied by a slight blue shift of the peak at short wavelengths when increasing the concentration of AgNO_3_ from 0.4 to 1.2 mM. The CD spectra of Ag NPs display, with the increase of AgNO_3_ concentration, the negative and positive Cotton effects at long wavelength exhibit redshifts that are corresponding to the changes of the absorption spectra. This phenomenon suggests that upon increasing the concentration of AgNO_3_, the aggregation level of the formed Ag NPs increases, which can be verified by TEM images. Figure 3c–e are the TEM images of Ag NPs prepared at pH 5.2 with various concentrations of AgNO_3_, showing that most of the particles are spherical and few are anisotropic in nature, with the average particle size of 12 ± 5 nm and the inter-particle distance of 1.1 ± 0.5 nm (Figure S4 in Supplementary Materials). With the increase of the concentration of AgNO_3_, the size of aggregates is gradually increased, which leads to the strengthening of the coupling between the neighboring Ag NPs in the aggregates. It should be noted that decreasing the pH value can also promote the aggregation of Ag NPs, enhancing the coupling of Ag NPs, and thus redshifting the absorption and CD bands (Figure S5 in Supplementary Materials).

The CD response of Ag NPs prepared at low pH is different from that of Ag NPs prepared at high pH (Figure 1b and Figure 3a). The pH value has a great influence on the composition of the mixed solution and the interaction between NaDC and Ag^+^. The decrease of pH of the solution causes NaDC molecules to be neutralized, generating HDC molecules. The formed HDC molecules can strongly interact with NaDC molecules due to the combination of hydrogen bonds and hydrophobic effects. The presence of hydrogen bonds between NaDC and HDC affects their interaction with the formed Ag NPs and thus has a great impact on the optical properties of Ag NPs.

**Figure 4:**
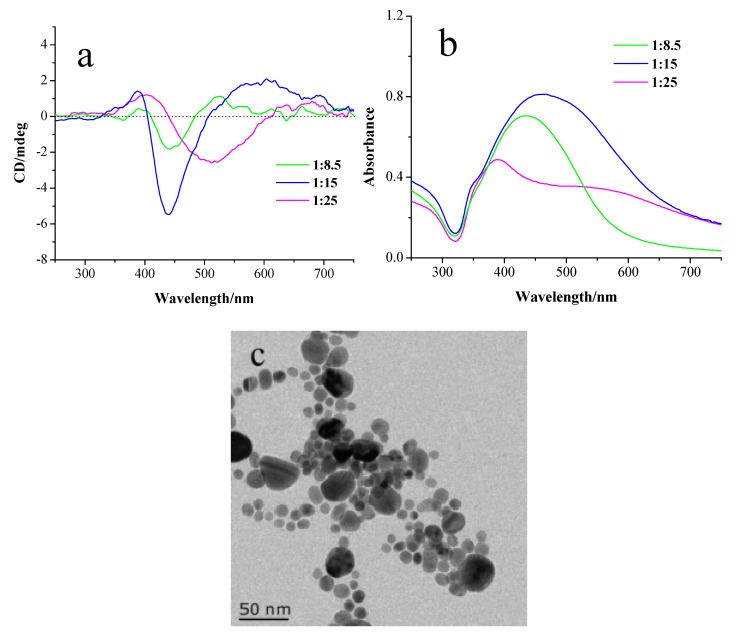
(**a**) CD and (**b**) UV-vis absorption spectra of Ag NPs prepared in the presence of NaDC and HDC with molar ratios of NaDC to HDC of 1:8.5, 1:15 and 1:25, respectively. (**c**) TEM images of Ag NPs prepared with molar ratio of NaDC to HDC of 1:15.

To verify this hypothesis, Ag NPs were prepared by adding NaBH_4_ to the mixed solution containing AgNO_3_, NaDC and HDC molecules. As shown in Figure 4a, the CD line shape of the Ag NPs prepared in this case is similar to that of Ag NPs prepared at a low pH value, suggesting that the mechanism of the plasmonic CD induction is identical. Their absorption spectra exhibit broad SPR peaks, indicating that strong plasmonic coupling occurs between Ag NPs (Figure 4b). Figure 4c is the TEM image of the Ag NPs prepared in this case, showing that Ag NPs are arranged in assemblies. TEM analysis indicates that the average particle distance between neighboring Ag NPs is around 1.0 ± 0.5 nm. Upon increasing the HDC concentration, the absorption peak redshifts and a new peak appears at a longer wavelength, suggesting that the plasmonic coupling is strengthened. The CD spectra show similar redshifts of the CD bands corresponding to the changes of the absorption spectra.

**Figure 5:**
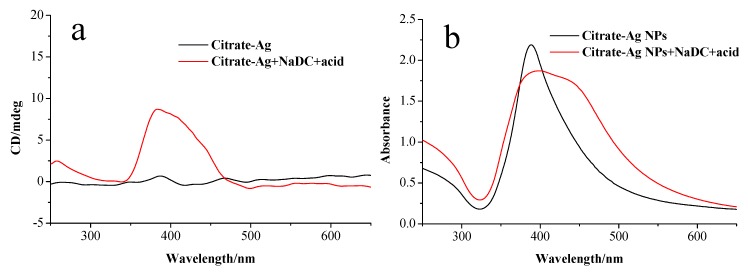
(**a**) CD and (**b**) UV-vis absorption spectra of sodium citrate-modified Ag NPs and the mixture of sodium citrate-modified Ag NPs, NaDC and acid.

From the above results, it can be seen that the presence of HDC is crucial for generating the split plasmonic CD lines. Our results show that this CD induction phenomenon relies on the synthesis condition. When citrate-modified Ag NPs were mixed with NaDC solution for half an hour and then acid was added, the produced Ag nanoparticles showed a positive Cotton effect at the SPR region in the CD spectra (Figure 5a). As shown in Figure 5b, the corresponding absorption spectrum of produced Ag NPs exhibits a shoulder at 430 nm in comparison with citrate-modified Ag NPs, indicating that the plasmonic coupling occurs between Ag NPs. The plasmonic CD response in this case is similar to that of NaDC-modified Ag NPs prepared by the ligand exchange reactions (Figure 2a). The differences in CD lines of Ag NPs (prepared by ex situ and in situ methods) at low pH indicate the importance of the interactions between Ag^+^, NaDC and HDC molecules. In the case of in situ preparation, the interactions between them facilitate the specific arrangement of NaDC/HDC molecules at hot spots between the formed Ag NPs, which is crucial for the induced split CD signals. This special oriented arrangement cannot be achieved in the case of ex situ preparation where Ag nanoparticles already exist.

Two distinct plasmonic CD features are observed for in situ prepared Ag NPs at different pH values, indicating that different mechanisms dominate the plasmonic CD induction. For both cases, aggregation occurs among Ag NPs that is mediated by NaDC molecules. For the synthesis at high pH, Ag NPs were assembled through the hydrophobic interaction between NaDC molecules that are on the surfaces of Ag NPs. However, the situation changes in the case of synthesis at low pH, where HDC molecules appear and interact with NaDC molecules via hydrogen bonds and hydrophobic interactions. The presence of the hydrogen bonds between NaDC and HDC molecules weakens the interactions between the COO– groups of NaDC and Ag NPs, varying the arrangement of NaDC/HDC molecules at gaps between Ag NPs. This can be verified by the varying inter-particle distances of Ag NPs prepared in the two cases (2.5 ± 1.1 nm for Ag NPs prepared at high pH and 1.1 ± 0.5 nm for those prepared at low pH). This clearly demonstrates that the inter-particle distance of Ag NPs prepared at low pH is short, suggesting a different arrangement of NaDC/HDC molecules at the gaps between the adjacent Ag NPs.

### 3.4. Mechanism of the Interactions between Chiral Molecules and Ag NPs

**Figure 6:**
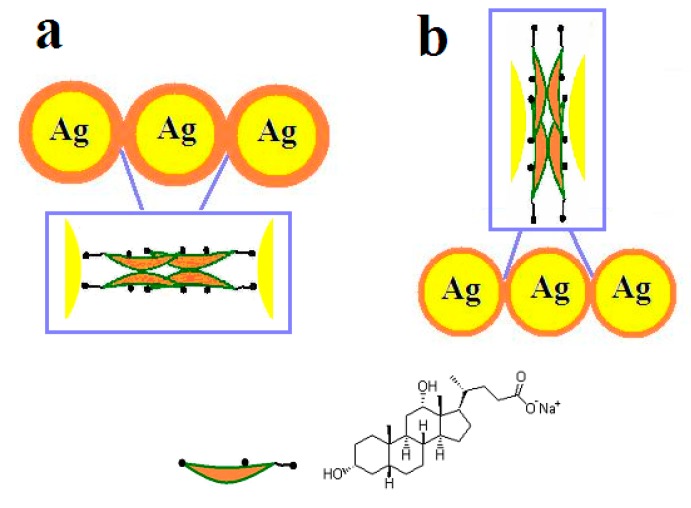
Illustration of the interaction modes between chiral molecules and Ag NPs: (**a**) at pH 7.0 and (**b**) at pH 5.2.

Based on the results of the experiments, a mechanism for the formation of Ag assemblies is proposed (Figure 6). For the synthesis at high pH, NaDC molecules are bonded to the surfaces of Ag NPs via COO– groups. The existence of NaDC molecules on the surfaces of Ag NPs facilitates the assembly of Ag NPs via hydrophobic interactions between NaDC molecules (Figure 6a). In the case of synthesis at low pH, the interactions between COO– groups of NaDC and Ag NPs are weakened due to the strong hydrogen bonding between the COO– group of NaDC and the COOH group of HDC. Consequently, in this situation OH groups of NaDC/HDC have the tendency to interact with Ag NPs (Figure 6b).

Govorov et al. predicted that the sign and strength of the plasmonic CD depends on the orientation of the molecular dipole with respect to the nanoparticle surface [31,32]. In addition to the orientations of chiral molecules, the inter-particle distances may also influence the strength and shape of the induced plasmonic CD. In the closely located metal nanoparticle system, the plasmonic coupling between them can be much stronger, which will result in the splitting of the CD lines due to the exciton coupling [35]. Hence, the mechanism for the distinct CD responses in the two cases would probably be related to the arrangements of the chiral molecules in the plasmonic hot spots and the inter-particle distances between Ag NPs. Compared with silver NPs prepared at high pH values, silver NPs prepared at low pH values exhibit strong plasmonic coupling between silver nanoparticles due to the short inter-particle distance, which results in the splitting of CD spectra.

## 4. Conclusions

We report herein a facile synthesis of NaDC-capped silver NPs by chemical reduction method in aqueous solution. The as-synthesized Ag NPs display two distinct plasmonic CD features, which are pH dependent. The arrangements of the capping agents in the plasmonic hot spots and inter-particle distances are crucial for the induced CD signals. By varying the pH values of the solutions, the arrangements of NaDC between Ag NPs in the aggregates can be controlled. For Ag NPs prepared at pH 7.0, the in situ and ex situ prepared NaDC-modified Ag NPs have similar CD signals. For in situ prepared NaDC-modified Ag NPs at low pH value, the CD signal is distinct from that of ex situ prepared NaDC-modified Ag NPs, revealing the importance of the interactions between Ag^+^, NaDC and HDC molecules in the reaction solution that facilitate the specific arrangement of NaDC/HDC molecules at hot spots after the reduction reaction.

## Supplementary Materials

The following are available online at http://www.mdpi.com/1996-1944/11/8/1291/s1. Figure S1: Size distribution of Ag NPs prepared at pH 7.0 (7.5 ± 2.5 nm, n = 400); Figure S2: Inter-particle distance distribution of Ag NPs prepared at pH 7.0 (2.5 ± 1.1 nm, n = 400); Figure S3: CD spectra of NaDC solutions with the concentrations of 0.4, 3.0, and 12.0 mM, respectively; Figure S4: Size distribution of Ag NPs prepared at pH 5.2 (12 ± 5 nm, n = 400) (a) and inter-particle distance distribution of Ag NPs prepared at pH5.2 (1.1 ± 0.5 nm, n = 336) (b); Figure S5: (a) CD and (b) UV-vis absorption spectra of Ag NPs prepared at pH 5.0 and 5.2.

## References

[1] Bjerneld E.J., Svedberg F., Käll M. (2003). Laser-induced growth and deposition of noble-metal nanoparticles for Surface-Enhanced Raman Scattering. Nano Lett..

[2] Wang X.J., Wang C., Cheng L., Lee S.T., Liu Z. (2012). Noble metal coated single-walled carbon nanotubes for applications in surface enhanced raman scattering imaging and photothermal therapy. J. Am. Chem. Soc..

[3] Zhang J.Z. (2010). Biomedical applications of shape-controlled plasmonic nanostructures: A case study of hollow gold nanospheres for photothermal ablation therapy of cancer. J. Phys. Chem. Lett..

[4] Wu Q.Z., Cao H.Q., Luan Q.Y., Zhang J.Y., Wang Z., Warner J.H., Watt Andrew A.R. (2008). Biomolecule-assisted synthesis of water-soluble silver nanoparticles and their biomedical applications. Inorg. Chem..

[5] Tamura M., Fujihara H. (2003). Chiral bisphosphine BINAP-stabilized gold and palladium nanoparticles with small size and their palladium nanoparticle-catalyzed asymmetric reaction. J. Am. Chem. Soc..

[6] Zhan P.F., Wang Z.G., Li N., Ding B.Q. (2015). Engineering gold nanoparticles with DNA ligands for selective catalytic oxidation of chiral substrates. ACS Catal..

[7] Wei J.J., Guo Y.J., Li J.Z., Yuan M.K., Long T.F., Liu Z.D. (2017). Optically active ultrafine Au–Ag alloy nanoparticles used for colorimetric chiral recognition and circular dichroism sensing of enantiomers. Anal. Chem..

[8] Zhang L., Xu C.L., Song G.X., Li B.X. (2015). Self-assembly of L-cysteine–gold nanoparticles as chiral probes for visual recognition of 3,4-dihydroxyphenylalanine enantiomers. RSC Adv..

[9] Xu Z., Xu L.G., Liz-Marzán L.M., Ma W., Kotov N.A., Wang L.B., Kuang H., Xu C.L. (2013). Sensitive detection of silver ions based on chiroplasmonic assemblies of nanoparticles. Adv. Opt. Mater..

[10] Abdulrahman N.A., Fan Z., Tonooka T., Kelly S.M., Gadegaard N., Hendry E., Govorov A.O., Kadodwala M. (2012). Induced chirality through electromagnetic coupling between chiral molecular layers and plasmonic nanostructures. Nano Lett..

[11] Yan W.J., Wang Y.L., Zhuang H., Zhang J.H. (2015). DNA-engineered chiroplasmonic heteropyramids for ultrasensitive detection of mercury ion. Biosens. Bioelectron..

[12] Kuzyk A., Schreiber R., Fan Z.Y., Pardatscher G., Roller E., Högele A., Simmel F.C., Govorov A.O., Liedl T. (2012). DNA-based self-assembly of chiral plasmonic nanostructures with tailored optical response. Nature.

[13] Yan W.J., Xu L.G., Xu C.L., Ma W., Kuang H., Wang L.B., Kotov N.A. (2012). Self-assembly of chiral nanoparticle pyramids with strong R/S optical activity. J. Am. Chem. Soc..

[14] Chen Z., Lan X., Chiu Y.C., Lu X.X., Ni W.H., Gao H.W., Wang Q.B. (2015). Strong chiroptical activities in gold nanorod dimers assembled using DNA origami templates. ACS Photonics.

[15] Shen X.B., Song C., Wang J.Y., Shi D.W., Wang Z.G., Liu N., Ding B.Q. (2012). Rolling up gold nanoparticle-dressed DNA origami into three-dimensional plasmonic chiral nanostructures. J. Am. Chem. Soc..

[16] Chu G., Wang X.S., Yin H., Shi Y., Jiang H.J., Chen T.R., Gao J.X., Qu D., Xu Y., Ding D.J. (2015). Free-standing optically switchable chiral plasmonic photonic crystal based on self-assembled cellulose nanorods and gold nanoparticles. ACS Appl. Mater. Interfaces.

[17] Bai P., Yang S., Bao W., Kao J., Thorkelsson K., Salmeron M., Zhang X., Xu T. (2017). Diversifying nanoparticle assemblies in supramolecule nanocomposites via cylindrical confinement. Nano Lett..

[18] Lukach A., Thérien-Aubin H., Querejeta-Fernández A., Pitch N., Chauve G., Méthot M., Bouchard J., Kumacheva E. (2015). Coassembly of gold nanoparticles and cellulose nanocrystals in composite films. Langmuir.

[19] Rezanka P., Zaruba K., Kral V. (2011). Supramolecular chirality of cysteine modified silver nanoparticles. Coll. Surf. A Physicochem. Eng. Asp..

[20] Wang R.Y., Wang P., Liu Y.N., Zhao W.J., Zhai D.W., Hong X.H., Ji Y.L., Wu X.C., Wang F. (2014). Experimental observation of giant chiroptical amplification of small chiral molecules by gold nanosphere clusters. J. Phys. Chem. C.

[21] Leroux F., Gysemans M., Bals S., Batenburg K.J., Snauwaert J., Verbiest T., VanHaesendonck C., Tendeloo G.V. (2010). Three-dimensional characterization of helical silver nanochains mediated by protein assemblies. Adv. Mater..

[22] Shemer G., Krichevski O., Markovich G., Molotsky T., Lubitz I., Kotlyar A.B. (2006). Chirality of silver nanoparticles synthesized on DNA. J. Am. Chem. Soc..

[23] Zhang H., Govorov A.O. (2012). Giant Circular dichroism of a molecule in a region of strong plasmon resonances between two neighboring gold nanocrystals. Phys. Rev. B.

[24] Ge′rard V.A., Gun’ko Y.K., Defrancq E., Govorov A.O. (2011). Plasmon-induced CD response of oligonucleotide-conjugated metal Nanoparticles. Chem. Commun..

[25] Roman-Velazquez C.E., Noguez C., Garzon I.L. (2003). Circular dichroism simulated spectra of chiral gold nanoclusters: A dipole approximation. J. Phys. Chem. B.

[26] Yao H., Miki K., Nishida N., Sasaki A., Kimura K. (2005). Large optical activity of gold nanocluster enantiomers induced by a pair of optically active penicillamines. J. Am. Chem. Soc..

[27] Goldsmith M.R., George C.B., Zuber G., Naaman R., Waldeck D.H., Wipf P., Beratan D.N. (2006). The chiroptical signature of achiral metal clusters induced by dissymmetric adsorbates. Phys. Chem. Chem. Phys..

[28] Gautier C., Bürgi T. (2006). Chiral N-isobutyryl-cysteine protected gold nanoparticles: Preparation, size selection, and optical activity in the UV−vis and Infrared. J. Am. Chem. Soc..

[29] Gautier C., Bürgi T. (2005). L-Glutathione chemisorption on gold and acid/base induced structural changes: A PM-IRRAS and time-resolved in situ ATR-IR spectroscopic study. Langmuir.

[30] Govorov A.O., Fan Z.Y., Hernandez P., Slocik J.M., Naik R.R. (2010). Theory of circular dichroism of nanomaterials comprising chiral molecules and nanocrystals: Plasmon enhancement, dipole interactions, and dielectric effects. Nano Lett..

[31] Govorov A.O. (2011). Plasmon-induced circular dichroism of a chiral molecule in the vicinity of metal nanocrystals. Application to various geometries. J. Phys. Chem. C.

[32] Nair P.P., Kritchevsky D., Setchell K.D.R. (1971). The Bile Acid: Chemistry, Physiology and Metabolism.

[33] Layani M.E., Moshe A.B., Varenik M., Regev O., Zhang H., Govorov A.O., Markovich G. (2013). Chiroptical activity in silver cholate nanostructures induced by the formation of nanoparticle assemblies. J. Phys. Chem. C.

[34] Zhu Z.N., Liu W.J., Li Z.T., Han B., Zhou Y.L., Gao Y., Tang Z.Y. (2012). Manipulation of collective optical activity in one-dimensional plasmonic assembly. ACS Nano.

[35] Guerrero-Martíneza A., Alonso-Gómezb J.L., Auguiéa B., Cidb M.M., Liz-Marzána L.M. (2011). From individual to collective chirality in metal nanoparticles. Nano Today.

