# Supplementary material for: Synthesis and Plasmonic Chiroptical Studies of Sodium Deoxycholate Modified Silver Nanoparticles
Source: Materials (Basel). 2018 Jul 26;11(8):1291. doi: 10.3390/ma11081291 (PMC6117651; doi:10.3390/ma11081291)
Supplement: Supplementary file 1 [file materials-11-01291-s001.pdf]

Synthesis and Plasmonic Chiroptical Studies of Sodium Deoxycholate Modified Silver Nanoparticles

Jing Wang*, Kai-Xuan Fei, Xin Yang, Shuai-Shuai Zhang, Yin-Xian Peng*

Supplementary Materials:

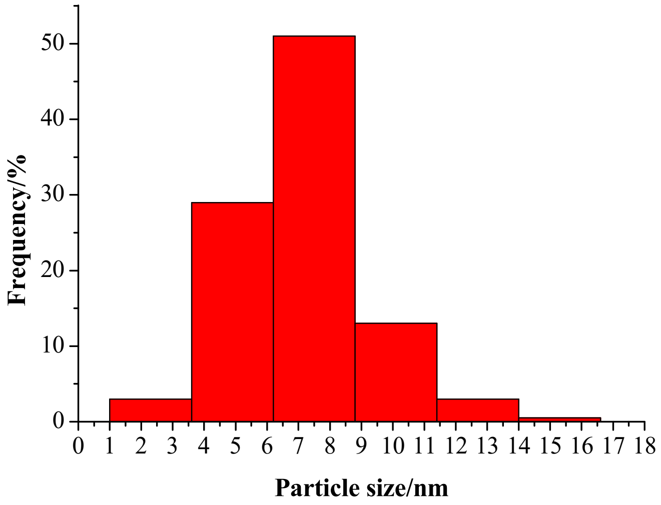

Figure S1. Size distribution of Ag NPs prepared at pH7.0 (7.5±2.5 nm, n=400). The analysis was performed using Nano Measurer 1.2 software. The statistics was done over 400 nanoparticles.

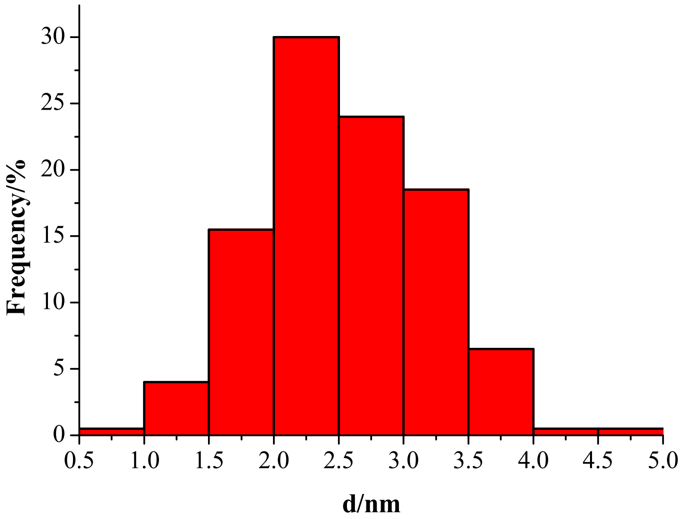

Figure S2. Inter-particle distance distribution of Ag NPs prepared at pH7.0 (2.5±1.1 nm, n=400) The analysis was performed using Nano Measurer 1.2 software. The statistics was done over 400 nanoparticles.

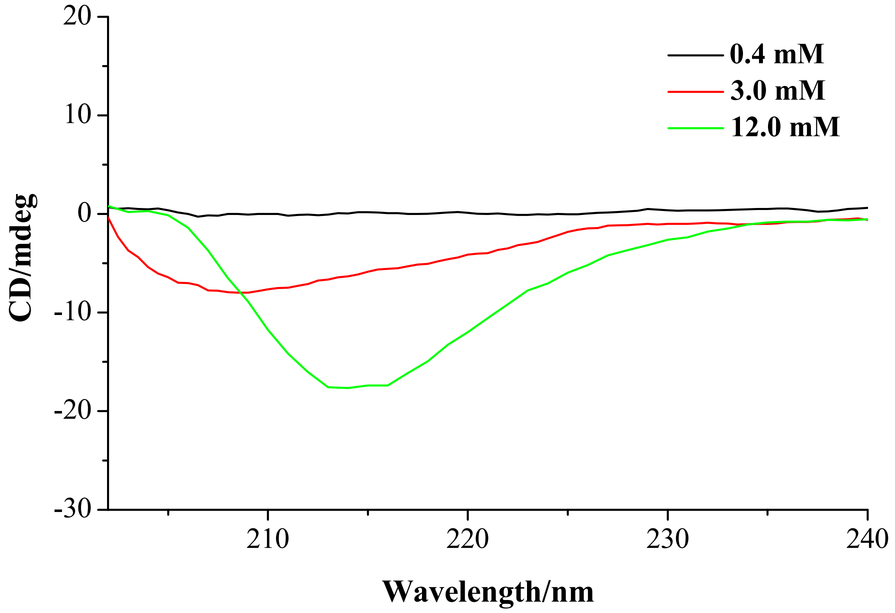

Figure S3. CD spectra of NaDC solutions with the concentration of 0.4, 3.0, and 12.0 mM, respectively.

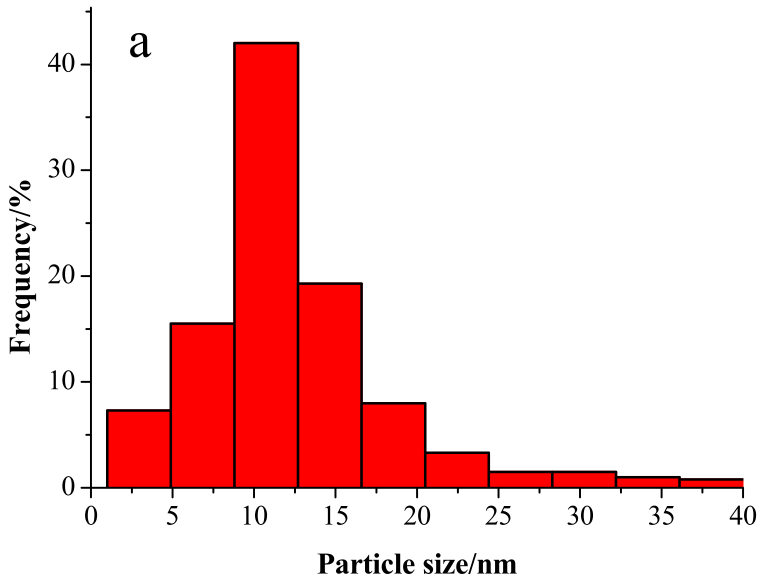

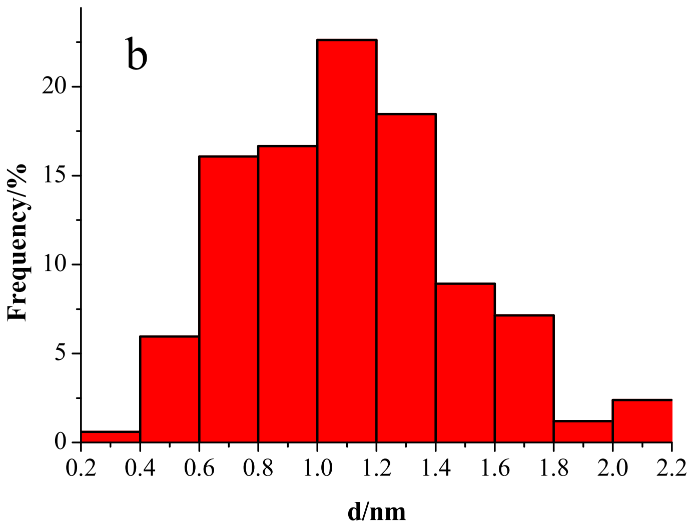

Figure S4. Size distribution of Ag NPs prepared at pH5.2 (12±5 nm, n=400) (**a**) and inter-particle distance distribution of Ag NPs prepared at pH5.2 (1.1±0.5 nm, n=336) (**b**). The analysis was performed using Nano Measurer 1.2 software.

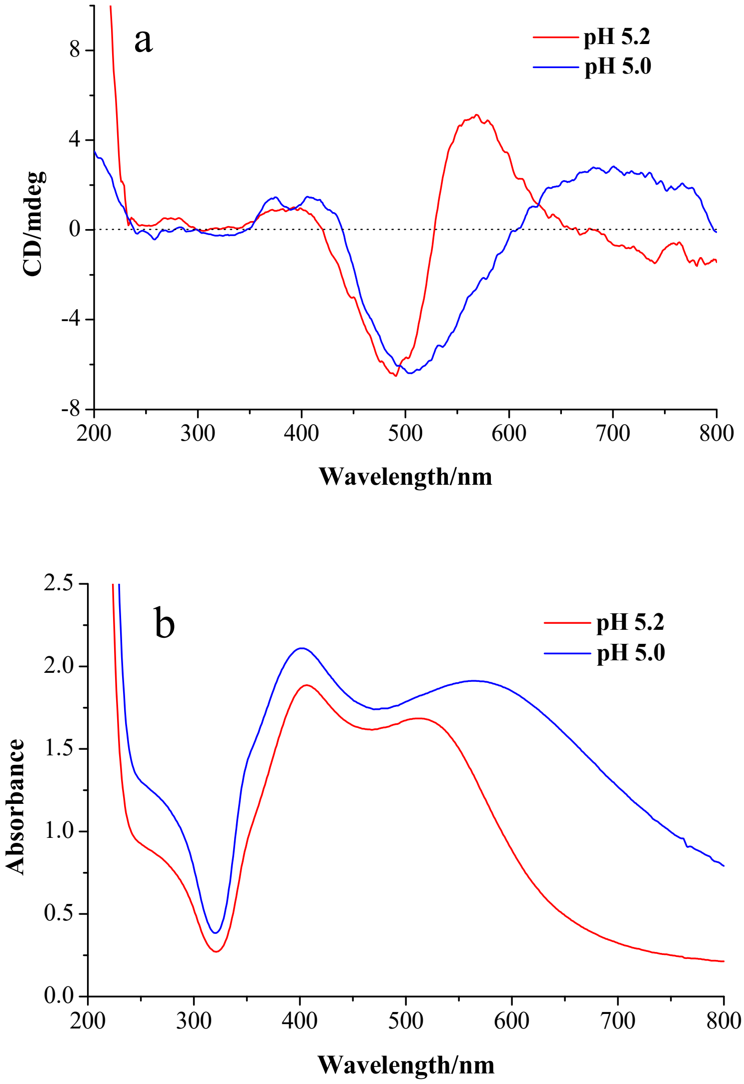

Figure S5. (**a**) CD and (**b**) UV-vis absorption spectra of Ag NPs prepared at pH 5.0 and 5.2.
